# A graph-based approach for designing extensible pipelines

**DOI:** 10.1186/1471-2105-13-163

**Published:** 2012-07-12

**Authors:** Maíra R Rodrigues, Wagner CS Magalhães, Moara Machado, Eduardo Tarazona-Santos

**Affiliations:** 1 Departamento de Biologia Geral, Universidade Federal de Minas Gerais, Av. Antonio Carlos 6627, Pampulha, Caixa Postal 486, 31270-910, Belo Horizonte, Brazil

## Abstract

**Background:**

In bioinformatics, it is important to build extensible and low-maintenance systems that are able to deal with the new tools and data formats that are constantly being developed. The traditional and simplest implementation of pipelines involves hardcoding the execution steps into programs or scripts. This approach can lead to problems when a pipeline is expanding because the incorporation of new tools is often error prone and time consuming. Current approaches to pipeline development such as workflow management systems focus on analysis tasks that are systematically repeated without significant changes in their course of execution, such as genome annotation. However, more dynamism on the pipeline composition is necessary when each execution requires a different combination of steps.

**Results:**

We propose a graph-based approach to implement extensible and low-maintenance pipelines that is suitable for pipeline applications with multiple functionalities that require different combinations of steps in each execution. Here pipelines are composed automatically by compiling a specialised set of tools on demand, depending on the functionality required, instead of specifying every sequence of tools in advance. We represent the connectivity of pipeline components with a directed graph in which components are the graph edges, their inputs and outputs are the graph nodes, and the paths through the graph are pipelines. To that end, we developed special data structures and a pipeline system algorithm. We demonstrate the applicability of our approach by implementing a format conversion pipeline for the fields of population genetics and genetic epidemiology, but our approach is also helpful in other fields where the use of multiple software is necessary to perform comprehensive analyses, such as gene expression and proteomics analyses. The project code, documentation and the Java executables are available under an open source license at http://code.google.com/p/dynamic-pipeline. The system has been tested on Linux and Windows platforms.

**Conclusions:**

Our graph-based approach enables the automatic creation of pipelines by compiling a specialised set of tools on demand, depending on the functionality required. It also allows the implementation of extensible and low-maintenance pipelines and contributes towards consolidating openness and collaboration in bioinformatics systems. It is targeted at pipeline developers and is suited for implementing applications with sequential execution steps and combined functionalities. In the format conversion application, the automatic combination of conversion tools increased both the number of possible conversions available to the user and the extensibility of the system to allow for future updates with new file formats.

## Background

In *silico* experiments are performed using a set of computer analysis and processing tools that are executed in a specific order. To automate the execution of these tools, they are usually organised in the form of a pipeline, so that the output of one tool is automatically passed on as the input of the next tool. In such a process, it is helpful to have tools that are designed in a way that guarantees the interoperability of all execution steps. The interoperability ensures that the output of a tool is processed by the subsequent tool even if the output format of the former does not match the input format of the latter. Aside from enabling task automation and data flow control, pipelines may be particularly advantageous if they allow an increasing number of possible operations offered to the user by combining different tools. For example, if we have four analysis tools: Blast [1], that finds sequence similarities for a DNA sequence; CLUSTALW [2], which aligns a set of sequences from different species; PHYLIP [3], which finds phylogenetic relationships from sequences of different species; and PAML [4,5], that infers sites under positive selection from a set of closely related sequences. In addition to their individual functionality, we can combine Blast, CLUSTALW and PHYLIP in a pipeline to find possible phylogenetic relationships for a DNA sequence. Alternatively, we can also compose a pipeline using Blast, CLUSTALW and PAML to infer sites under positive selection. Because the output of Blast is not compatible with the input of CLUSTALW, additional reformatting by ad hoc scripts is required to ensure the interoperability of the tools in the pipelines.

The traditional and simplest implementation of pipelines involves hardcoding the execution steps into programs or scripts. This approach leads to problems when pipelines need to be expanded, because the addition of new tools to such a pipeline is error prone and time consuming. An experienced programmer is needed to change the hard-coded steps of such pipelines to include new tools in the pipeline while maintaining bug-free functioning. These problems are a major concern not only for bioinformatics laboratories that want to continuously update their pipelines with new software developments, but also for those who want to consolidate open and cooperative systems [6,7].

An additional level of flexibility may be achieved by workflow management systems such as Taverna [8], Galaxy [9] and Pegasus [10] that are well suited for analysis tasks that are systematically repeated without changes in the course of execution, such as genome annotation [11,12] and the tasks registered at the myExperiment website [13]. Some workflow management systems also support dynamic execution of workflows, such as Kepler [14] and others [15], where dynamism occurs during the mapping and execution phases of the workflow’s life cycle [15] mainly for the instantiation of workflow components based on a high-level workflow description and data type compatibility verification. In these systems, the composition of the high-level workflow description is usually left to the user, which can either assemble his own group of tools or reuse an existing workflow description. However, in applications in which tools can be combined in different ways into a pipeline, it is difficult for the user to keep track of all possible combinations. This requires an automatic approach one level above execution, during the composition of the pipeline. This type of situation arises, for example, in format mapping, i.e., the conversion between software file formats that relies on a combination of conversion tools to map one format into another. Consider, for example, the following conversion system: tool*T*_*αβ*_ maps format *α* into format *β*, tool *T*_*βγ*_ maps format *β* into format *γ*, and *T*_*βδ*_ maps format *β* into *δ*. A workflow approach to implement such a conversion system requires the creation of five different workflows, one for each possible mapping (that is, *α*to *β*, *α* to *γ*, *α* to *δ*, *β* to *γ* and *β* to *δ*). In this case, to convert *α*into *β*, we would have *W*_*αβ*_(*T*_*αβ*_); to convert *α* into *γ*, we would have *W*_*αγ*_(*T*_*αβ*_*T*_*βγ*_); and to convert *α* into *δ*, we would have *W*_*αδ*_(*T*_*αβ*_*T*_*βδ*_). If a new conversion tool is added into this system, such as*T*_*δ∈*_, additional workflows are needed to implement the new functionality (in this case, *W*_*δ∈*_, *W*_*α∈*_ and *W*_*β∈*_). Without an automated process for composing workflows, these new workflows have to be created by users or by the system’s developers. In this case, the ideal solution would employ pipelines that are arranged “on the fly” in an automatic way, depending on the functionality required, instead of being statically programmed into a limited set of workflows.

In this paper, we propose a graph-based approach to design extensible pipelines. This approach is a solution for pipeline applications with multiple functionalities that require different combinations of steps in each execution. By automatically combining tools on demand into a pipeline according to the required functionality, it becomes unnecessary to specify every potential sequence of tools beforehand. For developers, this allows the implementation of low-maintenance bioinformatics pipelines. Also, users do not have to compose a pipeline for every different task, since all possible compositions are automatically available to the user. Extensibility is achieved once new tools are easily added to the pipeline system without any necessary change on the system’s code. In this way, the system can expand and the number of tools that it comprises can increase without the need for a specialised user with programming skills. To that end, we have developed special data structures and a pipeline system algorithm. We demonstrate the applicability of our approach by implementing a format conversion pipeline for the fields of population genetics and genetic epidemiology.

## Results

We represent the connectivity of pipeline components (programs) with a directed graph. If there is an edge *e* connecting two nodes *V*_1_ and *V*_2_ in a graph *G*, with *e* acting as the incoming edge of *V*_2_ and the outgoing edge of *V*_1_, then *e* is a component that receives input *V*_1_and that generates output *V*_2_. Pipeline components are programs (generally called tools), and they receive one or more inputs, perform some processing on these inputs and generate one or more outputs. Inputs and outputs are data file types. In terms of bioinformatics pipelines, graph edges are tools such as Blast and CLUSTAL, as well as tools that guarantee interoperability. Nodes represent the input and output formats required or generated by these tools (e.g., AASeq, NNSEq, and FASTA).

A path in the graph is any sequence of nodes connected by edges leading from one node to the next. This sequence of nodes can also be seen as a sequence of the edges that connect them. Therefore, a path through the graph connecting an input *X* to an output *Y * represents a pipeline, a sequence of tools, that must be executed to generate *Y * from *X*.

To implement the graph-based approach, we developed (i) a data structure called a Tool Registry, which contains information about the tools, such as the inputs that they receive, the outputs that they generate and the names of their executable file, among other information, and (ii) a pipeline system algorithm, which creates a graph representation of the tool registry, finds a path through the graph and generates an executable function-specific pipeline.

**Figure 1:**
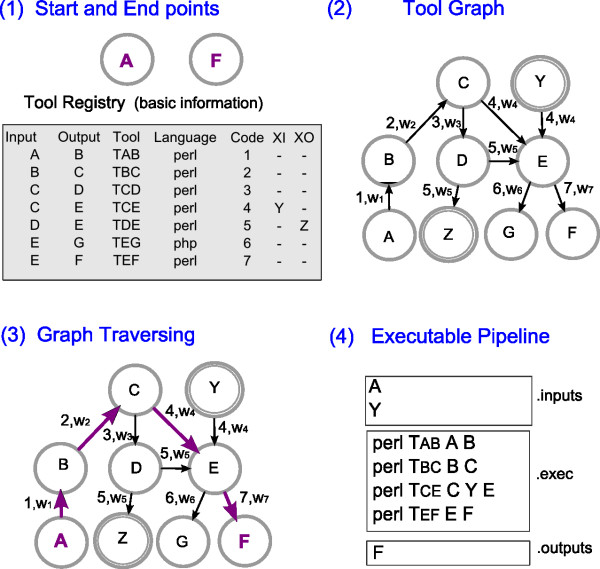
**Graphic representation of the pipeline system algorithm.** Graphic representation of the pipeline system algorithm. (**1**) algorithm inputs: start and end points, A and F (which are data formats), for a specific processing task, and the tool registry file; (**2**) directed graph built based on information from the tool registry, where regular nodes represent inputs and outputs, edges represent tools (denoted by their Code) and have a specific weight (*w*_*j*_), and double circled nodes represent input dependencies (XI) or secondary outputs (XO); (**3**) path through the graph connecting the start and end points, *P*_*A*,*F*_((*A*,*B*),(*B*,*C*),(*C*,*E*),(*E*,*F*)), generated by a graph-traversing procedure; (**4**) executable task-specific pipeline, which specifies the required inputs for the pipeline (file .inputs), the sequence of tools to be run (file .exec) and the output file (file .outputs).

The pipeline system algorithm is illustrated in Figure 1(1-4) and works generally as follows: (1) it receives as input the *start* and *end* points of the pipeline, which are, respectively, the original file to be processed and the desired resulting file, as well as the tool registry; (2) it builds a directed graph based on the registry file, using inputs and outputs as nodes and tools as edges connecting their respective inputs and outputs; (3) it applies a graph-traversing procedure to find a path through the graph connecting the *start* and *end* points, which represents the execution steps of a pipeline for a specific processing task; and (4) it returns this pipeline in an executable format. In Figure 1, letters represent data file types that are processed by bioinformatics tools. Although it is a simplification of real world cases, the illustration is intended to show how the connectivity among tools is represented in the graph, based on the descriptions on the Tool Registry.

In case there are alternative paths (or pipelines) available for the required processing task, the graph-traversing procedure selects the best one according to some criterion. We defined two alternative criteria for the best pipeline: performance, as measured by the speed of the pipeline, and input dependencies, according to which the selected pipeline is the one requiring the smallest number of input files. These criteria are called weight criteria (*wt*). The performance criterion in calculated on the basis of the tool’s response time for processing one or more input files of a specific size (see the Additional file 1 for more details on this calculation). The choice for one criterion or another can be presented to the system’s user, or the decision can be made by the system’s designer beforehand. We discuss the use of different selection criteria in Section Discus-sion. The components of our graph-based approach and the steps through the algorithm are explained in detail next.

We use the following notation to represent specific graph elements: *e*_*source*,*target*_, where *e* is the edge that connects a *source* node to a *target* node, and *P*_*start*,*end*_((*start*,*node*_1_),…,(*node*_*n*_,*end*)), where *P*_*start*,*end*_ is a path through the graph that begins at the *start* node and finishes at the *end* node passing by zero or more nodes.

### The tool registry

All information about the tools that are part of the pipeline system is stored in a Tool Registry. Each entry on the registry describes a particular tool with the following attributes (see Figure 1(1) for a partial representation): the input that it accepts (Input), which is a file type; the output that it generates (Output), which is also a file type; its executable file name (Tool); its programming language (Language); an identification number (Code); a list of extra input file types required to run it (XI or input dependencies); a list of secondary output file types generated by the tool (XO or subproducts); a performance measure indicating its average execution time (Performance); free text observations that the tool provider thinks the user should know in order to run it (Observations); and the provider’s name (Provider) and contact information (Contact). This information must be given by the tool provider before it is added as a new component of the pipeline system. A complete sample file is provided in the Additional file 1: Table S1.

New tool versions can be added to the registry with a new tool name. If the input and output file types from both versions are the same, the algorithm would find both tools as alternative paths and choose the one with best performance. If the input or output is different from the previous version, new format type names must be provided at the new version’s entry on the registry.

### Pipeline components

Pipeline components are programs or scripts that receive one or more inputs, perform some processing on these inputs and generate one or more outputs. To generate executable pipelines automatically, we define a specific format for the command line calls used to invoke the pipeline components: <Tool> <Input> [Input1.n] <Output> [Output1.n]

Here, Input and Output are the tool’s parameters stored in the tool’s entry in the Tool Registry. Parameters in square brackets are optional and correspond to the tool’s extra inputs and secondary outputs. We discuss an extension to this command line format in Section Discussion.

### Pipeline system algorithm

To generate an executable pipeline for a specific functionality, such as converting data file *A* to data file *F*, our pipeline system algorithm builds a directed graph on the basis of the tool registry, and it finds a path through this graph using the original input to be processed (*A*) as *start* point and the desired output (*F*) as the *end* point. This path represents a pipeline where the sequence of edges in the path is the sequence of tools to be run. This process is illustrated in Figure 1 (and a formalisation of the algorithm can be found on the Additional file 1).

The algorithm receives as input the *start* and *end* points, the tool registry file (*toolRegistry*) and the weight criterion to be applied to the graph edges (*wt*). It starts by building a directed graph *G* based on the information in the tool registry file. This process is accomplished by taking each entry in the tool registry, represented by *E*_1_,…,*E*_*j*_, and parsing it into a tuple *E*_*j*_(*i*,*o*,*t*,*l*,*c*,*XI*,*XO*,*f*,*b*,*r*,*e*), where each element corresponds to a field (or column) in the tool registry. It then adds the input and output information, *E*_*j*_[*i*] and *E*_*j*_[*o*], as nodes in graph *G* and the tool’s name, *E*_*j*_[*t*], as an edge connecting its respective input and output. If the input and output file types of a specific tool are the same, an edge is created in the same way as before. In this case, edge’s source and target nodes will be the same. Provided that a tool to trim or filter files of the same type, generating an output file with different content but of the same file type as the input, is included in the Tool Registry, our solution allows adding tools that perform these tasks. To each edge, we assign a weight *w*_*j*_ that is calculated according to the chosen criterion (*wt*) for selecting among multiple paths. If *wt* is *performance*, then *w*_*j*_ is the performance measure *E*_*j*_[*f*]; if *wt* is *dependencies*, then *w*_*j*_ is the length of the input dependencies list for that tool, *length*(*E*_*j*_[*XI*]).

After that, the same process is repeated for adding to the graph both the list of input dependencies (*E*_*j*_[*XI*]) and the list of secondary outputs (*E*_*j*_[*XO*]) for all tools. The only difference is that the graph edges connecting extra inputs and outputs to other nodes receive a symbolic zero weight, since they do not account for any processing task. Also, if a node is equal to an extra input or to an extra output already found in *G*, an alias is created (a numerical index) so that these extra input or output nodes can be added to the graph (such as *A*_1_ if *G* already contains a node *A*).

With the tool registry represented as a directed graph, the algorithm then searches for a path (*P*) to connect the *start* and *end* points (such as data files *A* and *F*, in Figure 1). This process is accomplished using a graph-traversing shortest path procedure that implements Dijkstra’s shortest path algorithm (we have tested the implementation of other shortest path algorithms such as Bellman-Ford, but they did not show any difference in performance). If a path exists, it represents the sequence of tools that need to be run to generate the desired output. This process is illustrated in Figure 1(3), where the path connecting the *start* and *end* points *A* and *F* is *P*_*A*,*F*_((*A*,*B*),(*B*,*C*),(*C*,*E*),(*E*,*F*)), and its corresponding tool path is *P*_*A*,*F*_((*T*_*AB*_),(*T*_*BC*_),(*T*_*CE*_),(*T*_*EF*_)). If no path is found, then there is no available pipeline for the required processing task. On the other hand, if there is more than one possible path connecting the start and end points, the shorted path procedure chooses the path with the smallest sum of its composing edges’ weights. As mentioned before, this process entails selecting the path that will result in a pipeline composed of the best performing scripts (when the performance criterion is used) or requiring less user intervention (if the dependency criterion is used).

After finding the pipeline for the required processing task, the algorithm generates an executable version of this pipeline. This process is illustrated in Figure 1(4). The executable version indicates the inputs required to run the pipeline (file .inputs), the command line call for each tool (file .exec), and the outputs that are generated (file .outputs).

Required inputs (which we call list *LI*) include, in addition to the original file to be processed, the input dependencies that might exist for each tool that will run in the pipeline. For example, of all the tools in *P*_*A*,*F*_((*T*_*AB*_),(*T*_*BC*_),(*T*_*CE*_),(*T*_*EF*_)), *T*_*CE*_ (or *E*_4_[*t*]) is the only one with an extra input file *E*_4_[*XI*] = {*Y*}. This information is extracted from the Tool Registry. Thus, *LI* = {*A*,*Y*}. Similarly, the output files of the pipeline (which we call list *LO*) include, in addition to the desired output file, any secondary outputs that might be generated by each tool in the pipeline. For example, in *P*_*A*,*F*_, none of the tools has an extra output file; in this case, *LO* = {*F*}. These lists of inputs and outputs are used to generate the files .inputs and .outputs.

In the file .exec, tools are invoked by a command line call with the following format (see Section Pipeline Components):

(1) $$\begin{array}{c} \;E_{n}[l]\;E_{n}[t]\;E_{n}[i]\;E_{n}[\mathrm{XI}[1\mathrm{.k}]]\\ E_{n}[o]\;E_{n}[\mathrm{XO}[1\mathrm{.k}]] \end{array}$$

where *E*_*n*_[*l*] is the programming language call, *E*_*n*_[*t*] is the executable name, *E*_*n*_[*i*] is the input, and *E*_*n*_[*o*] is the output for all *E*_*n*_[*t*]∈*P*. The parameters *E*_*n*_[*XI*[1*.k*]] and *E*_*n*_[*XO*[1*.k*]] are optional and represent extra inputs and secondary outputs for each tool.

### Running the executable function-specific pipeline

The executable function-specific pipeline in the .exec file can be run as a shell file or incorporated into another application as a set of system calls. The user just needs to provide the required input files (in file .inputs). Tools in the .exec file execute locally on the same machine. Since our pipeline design approach focuses on pipeline composition instead of execution, we have adopted a simpler execution mechanism. For error control, we provide a .err file, which stores error messages generated during the execution of the function-specific pipeline. Quality control procedures for input data must be implemented within each independent tool by its provider, since each processing task or data format will have its own requirements. This type of setup helps to maintain the system’s modularity and extensibility.

For a broader application that requires a more user-friendly interface, the three files generated by the pipeline system algorithm can be easily incorporated into a graphical interface to create an interactive pipeline. An example of an interactive pipeline system written in PHP is provided at the project’s website and is described in Section A format conversion pipeline application. This web-based system reads the .inputs file and presents to the user an upload page requiring all inputs specified in this file. When all required inputs are uploaded into the system, it executes all system calls in the file .exec, in order. After the last system call is finished, the interface system reads the file .outputs and presents the user with a link to each of the output files specified in the list. A similar procedure can be used to incorporate the pipeline system into a standalone application.

### Adding new tools

To add a new tool to the pipeline system, a new entry must be added in the Tool Registry containing the information about the new tool, therefore, no programming is required. This update can be performed directly by the tool’s developer or by the system’s administrator upon request from the tool’s developer that, in this case, must send all the required information about the tool. The ordinary user sees only the final result and the next time that he uses the pipeline system, the new tool’s functionality will be considered as part of the pipeline composition. This is possible since our pipeline system algorithm automatically and on demand generates the tool graph including this information. The only requirement for adding a tool to a pipeline system implemented with our algorithm is that it must follow the command line format described earlier in Section Pipeline components. Also, if the new tool requires a file type that is not already specified in the pipeline system, it is recommended that the developer provides a sample of such an input file so that a benchmark can be run to determine the tool’s performance.

### A format conversion pipeline application

We applied our graph-based approach to implement an automatic pipeline system for data format mapping in the fields of population genetics and genetic epidemiology. These fields, and others such as gene expression and proteomics analyses, require a specific set of data analysis procedures that use several different software packages [16-18]. Since most of these programs are not compatible in terms of accepted input and output formats, solutions to allow interoperability are required. We proposed elsewhere [19] a conversion pipeline to solve this interoperability problem in the context of DNA re-sequencing data. This conversion pipeline is composed of a set of scripts that convert one specific format to another. By combining such specialised scripts in a pipeline, we increase the number of possible conversions that are available to the user. In the [19] pipeline, however, possible combinations of scripts are hard-coded into the system, and thus, extension with new tools is costly because of the need for an experienced programmer to alter all of the pipeline code. To avoid this problem, we applied our graph-based approach to implement a dynamic version of this conversion pipeline. By combining the conversion scripts on demand into a pipeline based on the specific conversion required, it becomes unnecessary to specify beforehand the sequence of scripts for performing every possible conversion. We also added new tools to the original pipeline to increase the scope of its functionality.

Currently, our format conversion pipeline handles data formats that are compatible with the following software: PolyPhred (for polymorphism identification from aligned raw sequences reads), PHASE (to infer chromosome phase), DnaSP (for general population genetics analysis), Structure (for population structure inferences), Sweep (for natural selection inferences), Haploview (for linkage disequilibrium analysis) and R-based tools for population genetics and genetic epidemiology such as HierFstat (for inferences about population structure) (more information about these software packages is available as Additional file 1). The pipeline also handles general purpose file formats such as SDAT, NEXUS and PrettyBase. It comprises 15 conversion tools implemented in Perl, which allow for 26 possible format conversions.

**Figure 2:**
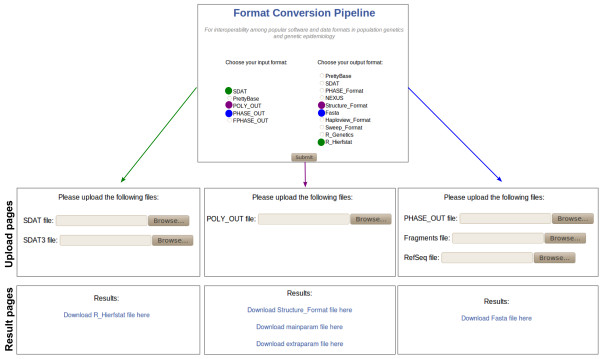
**Web interface for our format conversion pipeline.** Three scenarios are depicted: a conversion from SDAT format to R HierFstat format (denoted in green); a conversion from the PolyPhred output format to the Structure input format (denoted in purple); and a conversion from the PHASE output format to DnaSP input format or Fasta format (denoted in blue).

**Figure 3:**
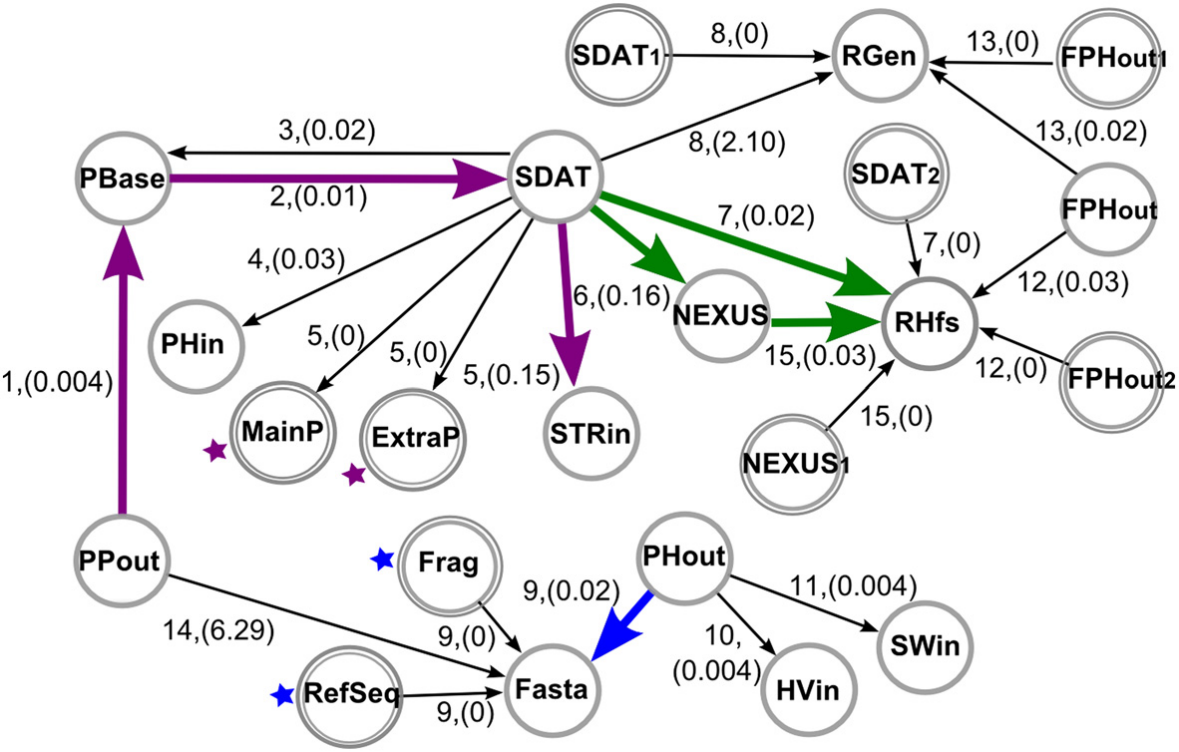
**Tool Graph for our format conversion pipeline system.** Nodes are popular data formats from population genetics and genetic epidemiology. Edges are labelled with the conversion tool’s Code and have an associated weight (represented in round brackets) indicating the tools’ performance.

To make the format conversion pipeline interactive and available online, we implemented our pipeline system algorithm as part of the web interface shown in Figure 2. Its website is hosted at http://pggenetica.icb.ufmg.br/divergenome/pagina/dynamicpipeline/tools.php. The algorithm is invoked after the user selects the input format and desired output format (Figure 2, top). Examples of the file formats are available at our re-sequencing pipeline website ( http://www.cebio.org/pipelineldgh/). The tool registry for this application is shown partially on Table 1 and in more detail in the Additional file 1: Table S1. The registry is used by the pipeline system algorithm to generate the graph in Figure 3. Here, graph nodes represent data formats, and edges represent the conversion tools’ codes with their corresponding weights. In our application, we used the performance criterion for selecting the best path among alternatives. Thus, edge weights are the performance measures that are specified for each tool in the tool registry. Note that extra inputs and outputs are represented by double circled nodes, as before, and they are renamed by adding a numerical index to their format name, in case they already appear in the graph (such as *SDAT*_1_, *SDAT*_2_ or *NEXUS*_1_). The weights of the latter incoming or outgoing edges are set to 0 since they do not account for any processing task. To demonstrate the functionalities provided by our automatic pipeline approach, we present three different potential usage scenarios.

**Table 1:** Tool Registry example for format conversion pipelines

**Input**	**Output**	**Tool**	**Language**	**Code**	**XI**	**XO**	**Performance**
PolyPhred	PrettyBase	PolyPhred2PrettyBase.pl	perl	1	-	-	0.004
PrettyBase	SDAT	PrettyBase2SDAT.pl	perl	2	-	-	0.01
SDAT	StructureFormat	SDAT2Structure.pl	perl	5	-	mainparam,	0.15
						extraparam	
SDAT	RHierfstat	SDAT2Rhierfstat.pl	perl	7	SDAT	-	0.02
PHASEOUT	Fasta	Phase2Fasta.pl	perl	9	Fragments,	-	0.02
					RefSeq		

Columns Input and Output are file formats that are accepted and generated by a conversion tool; Tool and Language are the conversion tool’s name and its programming language; Code is the identifier of the tool; XI is the list of extra input files required for the tool’s execution; XO is the list of extra output files that is generated by the tool; and Performance is a measure related to the tool’s execution time. Other information not represented on this table can be found in the Additional file 1: Table S1.

#### SDAT to R-HierFstat

First, let us suppose that, in a population genetics study, a researcher downloaded a dataset in SDAT format, containing a matrix of genotypes per sample and locus, and now the researcher wants to perform an analysis with the R package HierFstat to compute and test fixation indices for any hierarchical level of population structure. Since the SDAT format is not a valid input for HierFstat because the latter requires additional population information, the user needs to convert the SDAT format. To perform this conversion, the user chooses the two file formats of interest on the web interface shown in Figure 2 (top, in green), SDAT and RHierfstat. As visualised in the graph in Figure 3 (green arrows), there are two possible paths for this conversion: *P*_1_((*SDAT*,*RHfs*)) or *P*_2_((*SDAT*,*NEXUS*), (*NEXUS*,*RHfs*)). From these, the first path is chosen since its sum of edge weights (0.15) is smaller than that of the second path (0.36), meaning that the pipeline corresponding to the former path is the fastest. The tool path for this selected path is *P*_1_((*SDAT*2*Rhierfstat.pl*)) (see Table 1, line 4).

The tool path *P*_1_is used by the pipeline system algorithm to generate the executable pipeline for the specific conversion, as described in Section Pipeline system algo-rithm. The three output files of the executable pipeline are shown in Table 2 (top). They are handled internally by the system and the users see only the final web interface. Upload boxes for each required input are built into the interface based on the .inputs file (Figure 2, left). Each SDAT file corresponds to different populations that should be included in the study. Although for simplicity we show only one extra SDAT file for the tools converting from SDAT to RHierFstat in Table 1 and Figure 2, in practice these tools currently accept up to five populations. After these input files are uploaded, the interface reads the .exec file, runs it as a shell file, and presents the output files in .outputs as links for the user to download from.

**Table 2:** Executable pipelines for three usage scenarios

** File**	**Code**
** SDAT to R HierFstat**	
.inputs	SDAT02
	SDAT202
.exec	perl SDAT2Rhierfstat.pl SDAT02 SDAT202 RHierfstat02
.outputs	RHierfstat02
** PolyPhred output to Structure**	
.inputs	PolyOut01
.exec	perl PolyPhred2PrettyBase.pl PolyOut01 PrettyBase01
	perl PrettyBase2SDAT.pl PrettyBase01 SDAT01
	perl SDAT2Structure.pl SDAT01 StructureFormat01 mainpar01 extrapar01
.outputs	StructureFormat01
	mainparamt01
	extraparam01
** Phase to Fasta**	
.inputs	PHASEOUT03
	Fragments03
	RefSeq03
.exec	perl Phase2Fasta.pl PhaseOut03 Fragments03 RefSeq03 Fasta03
.outputs	Fasta03

Executable pipelines for file-format conversions: (top) SDAT format to R Hierfstat input format; (centre) PolyPhred output format to Structure input format; and (bottom) software PHASE output format to Fasta format. In practice, input and output files handled by the pipeline system are renamed to include a timestamp identifier of each specific pipeline (such as numbers 01, 02 and 03 above). This guarantees that inputs and outputs stored in the system are unique for each dynamically generated pipeline.

#### PolyPhred to structure

In the second scenario, the software package Phred-Phrap-Consed-PolyPhred is used for variation screening and one follow up analysis is to infer population structure using the program Structure. This may be useful, for example, if a set of linked chromosome regions have been re-sequenced in a set of individuals, and the linkage model of Structure [20] is intended to be used to explore the population structure of this genomic region. The output and input files generated and accepted by these two software programs are not compatible, and thus, the user needs to convert the output of the end-line software PolyPhred, containing individual genotypes and, into the input for Structure. To do so, the user chooses the two file formats of interest on the web interface shown in Figure 2 (top, in purple), PolyOut and Structure_Format.

The path found by our algorithm, shown in purple arrows in Figure 3, is *P*_3_((*PPout,PBase*), (*PBase, SDAT*), (*SDAT, STRin*)), which corresponds to the tool path *P*_3_((*PolyPhred2PrettyBase.pl*), (*PrettyBase2SDAT.pl*), (*SDAT2Structure.pl*)) (see Table 1). The executable pipeline that is generated by our algorithm for the specific conversion and implementation of this tool path is shown in Table 2 (centre). Note that this pipeline requires only one input file but generates three output files, which are displayed in Figure 2 (centre). This is because the end-line tool *SDAT2Structure.pl* in *P*_3_ has two extra output files (*mainparam* and *extraparam*), which are necessary to run the program Structure.

#### PHASE to DnaSP

For the third scenario, we take the fact that, in population genetics studies, it is common to run the software PHASE to infer haplotype phase and then perform general population genetics analysis with the program DnaSP. Since the input and output of these software tools are not compatible, the user needs to convert the output of PHASE, containing phased polymorphic sites, to the input format for DnaSP, a Fasta file. This conversion can be accomplished by selecting the two file formats of interest on the web interface shown in Figure 2 (top, in blue). The path found by our algorithm that connects PHASE output format (PHout) and Fasta format is depicted in Figure 3 with blue arrows and is formalised as *P*_4_((*PHoutFasta*)). Its corresponding tool path is *P*_4_(*Phase*2*Fasta.pl*) (see Table 1, line 5). The executable pipeline generated by the algorithm for the specific conversion is shown in Table 2 (bottom). It requires three input files, (the *PHASE output*, *Fragments*, and *RefSeq*), and generates one output file, (the *Fasta* file). This is because the tool *Phase2Fasta.pl* has two extra input files, which are necessary to build the new Fasta sequence (see [19] for details).

## Discussion

Building extensible systems is essential to ensure that new tools and data formats can be used with existing systems. This principle applies to the design of pipelines, a common task in most bioinformatics laboratories. Here, we propose a graph-based approach to this view of extensible pipelines, in contrast to traditional *ad hoc* pipeline designs.

Our approach is suitable for sequential pipelines in which each execution requires different combinations of steps through the pipeline. We have shown one such pipeline application for format mapping for population genetics and genetic epidemiology analyses. This pipeline provides 26 possible format conversions that originate from the combination of 15 independent conversion tools. By combining these scripts on demand into a pipeline according to each required conversion, it is not necessary to specify every possible combination of scripts beforehand. Moreover, with the graph-based implementation, new format conversion tools can be easily incorporated, and the system can stay updated. For instance, our group is developing conversion tools compatible with the SAM formats created by the 1000Genomes Project team [21]. Our approach also allows prompt integration of third party conversion tools developed by collaborators or available in public software repositories. The process of third-party adding new tools to the system was tested with the tools *SDAT2Rgenetics.pl*, *SDAT2Rhierfstat.pl* and *SDAT2NEXUS.pl* which were later incorporated by different group members of our laboratory.

Notably, when planning the addition of a new tool to the pipeline system, it is possible to take advantage of graph properties such as node connectivity to maximise the number of new functionalities. For example, taking our application graph in Figure 3, it is clear that if you develop a conversion tool that maps formatX into the NEXUS format, you gain only one additional conversion when adding this tool to the system (that is, formatX to RHfs). On the other hand, if you develop a conversion tool mapping formatX into SDAT format, you gain 6 additional conversions (that is, formatX to PBase, PHin, STRin, NEXUS, RHfs and RGen). We provide a java program in the project’s website ( http://code.google.com/p/dynamic-pipeline/) to help with this analysis.

In contrast, to implement the same format conversion pipeline with a workflow management system [8,9], it would be necessary to create a separate workflow for each possible inter-format conversion. Also, these workflow management systems are more frequently used in genomic sciences and focus on workflow execution, while their users (or their bioinformatics assistants) have to select and combine their specific components. Another example is the Pegasus framework [10], which is very robust on managing workflow execution but does not address the problem of automatic composition. Differently, our approach has been developed keeping in mind users who may not be necessarily bioinformatics experts and who require assistance on the combination of tools to be used in a specific analysis. For this purpose, our approach incorporates pipeline automatic composition as a conceptual and operational instrument to facilitate its use.

Similar work on automatic service composition, such as Magallanes [22] and Bio-jETIi [23] also focus on linear workflows and components with basic interfaces (such as tools accepting only file inputs and outputs). However, the main difference is that they present a different implementation for the automatic composition problem, not graph-based, and their approaches consider web services to compose the workflows, without performance information. The automatic pipeline approach, on the contrary, integrates *ad hoc* bioinformatics tools or scripts, in our case format conversion tools for population genetics or genetic epidemiology, with an associated performance measure that is used to select among possible alternative pipeline executions. Another difference regards the generation of an executable pipeline. In the case of Magallanes, it does not generate an executable workflow but only a model to be instantiated with web services by workflow management tools. Similarly, in Bio-jETI automatic service composition starts only after the user has assembled a high-level workflow specification manually through a graphical interface.

At present, our system can only perform automatic composition based on computer-measurable metrics, such as processing time, memory usage, and accuracy, among others. This is to guarantee the composition of a pipeline without user intervention. However, our approach has the potential to accommodate a user-centered choice, either based on his preferences or the context of his analysis. To implement that, instead of automatically selecting a pipeline among alternatives, our algorithm can be modified to present these alternative pipelines to the user, which can then select the best one.

A current limitation of our approach is that it cannot yet be used for automatically designing pipelines that require the execution of parallel steps because it focuses on the problem of finding alternative sequential steps to achieve a particular aim. However, adjusting our algorithm to support the second type of pipeline is straightforward. This can be done by taking alternative paths through a tool graph with overlapping edges as single pipelines where non-overlapping steps are executed concomitantly. Therefore, if there are three edges connecting nodes A and B, that is, three different tools processing file type A into B, the parallel algorithm would select all three tools to be executed at the same time.

For future development, we are studying an extension to the current algorithm to allow the inclusion of software tools that require specific command line parameters, such as strings and thresholds. Currently, pipelines are created with a set of tools that each use a standard command line interface that allows for the specification of one or more input files and one or more output files. We are working on a XML implementation of the Tool Registry to incorporate definitions of different classes of input parameters for the tools, such as files (the one currently accepted), strings and numerical values. This extension will allow the incorporation of bioinformatics tools that require different types of parameters, and general bioinformatics programs available in public repositories such as BioPerl and BioJava. We will consider current work on semantic service description, such as OWL-S [24] and the Web Services Description Language (WSDL) [25] to develop the XML-based Tool Registry. Finally, although here we have focused on applications that are composed of software tools, our graph-based approach could also be used to create pipelines that are composed of workflows or web services. This would only require a modification of the function that generates the executable pipeline so that it generates executable code that is compatible with each specific technology.

## Conclusions

Our graph-based approach enables the automatic creation of pipelines by compiling a specialised set of tools on demand, depending on the functionality required. It allows the implementation of extensible and low-maintenance pipelines and contributes towards consolidating openness and collaboration in bioinformatics systems. It is targeted at pipeline developers and is suited for implementing applications with sequential execution steps and combined functionalities. The algorithm serves as an alternative to workflow systems since it generates pipelines automatically without living the composition to the end-user. We have shown that this is the case for format conversion applications, in which the automatic combination of conversion tools increases the number of possible conversions available to the user and increases the extensibility of the system to allow for future updates with new file formats. Future developments will include an adaptation of our pipeline algorithm to enable the generation of pipelines with parallel steps and to allow the inclusion of tools that require external parameters. Extensions are also possible to generate executable code that is compatible with specific technologies, such as web services and workflows.

## Methods

The pipeline system algorithm was implemented in Java and we used the package jgraphT to implement the graph-related functions. The format conversion tools that compose the format conversion pipeline application were implemented in Perl. The format conversion pipeline’s web interface was implemented in PHP. The system has been tested on Linux and Windows platforms. Only Java is required for running the algorithms; for using the PHP web interface code, a web server such as Apache is required.

## Competing interests

The authors declare that they have no competing interests.

## Authors’ contributions

MRR conceived and developed the pipeline system algorithm, the java executables and the format conversion pipeline’s web interface. WCSM contributed to the design of the pipeline system algorithm and developed the format conversion tools used in the pipeline application. MM benchmarked and tested the tools composing the format conversion pipeline application. ETS supervised the project. MRR, WCSM, and ETS wrote the manuscript. All the authors read and approved the final manuscript.

## Supplementary Material

Additional file 1 **Supplementary Information.** This document provides additional information on the performance measure, the pipeline system algorithm, the list of tools in the pipeline application, and the complete Tool Registry for the Format Conversion Pipeline. Click here for file

## References

[B1] Altschul S Gish W Miller W Myers E Lipman D Basic local alignment search tool J Mol Biol 1990 215 403 410 2231712 10.1016/S0022-2836(05)80360-2

[B2] Thompson JD Higgins DG Gibson TJ CLUSTAL W: improving the sensitivity of progressive multiple sequence alignment through sequence weighting, position-specific gap penalties and weight matrix choice Nucleic Acids Res 1994 22 4680 10.1093/nar/22.22.4673 PMC308517 7984417

[B3] Felsenstein J PHYLIP – Phylogeny Inference Package (Version 3.2) Cladistics 1989 5 164 166

[B4] Yang Z PAML: a program package for phylogenetic analysis by maximum likelihood Comput Appl Bio Sci 1997 13 555 556 9367129 10.1093/bioinformatics/13.5.555

[B5] Yang Z PAML 4: a program package for phylogenetic analysis by maximum likelihood Mol Biol Evol 2007 24 1586 1591 10.1093/molbev/msm088 17483113

[B6] Stein L Creating a bioinformatics nation Nature 2002 417 6885 119 120 10.1038/417119a 12000935

[B7] Kaye J Heeney C Hawkins N de Vries J Boddington P Data sharing in genomics - re-shaping scientific practice Nat Rev Genet 2009 10 331 335 10.1038/nrg2573 19308065 PMC2672783

[B8] Hull D Wolstencroft K Stevens R Goble C Pocock M Li P Oinn T Taverna: a tool for building and running workflows of services Nucleic Acids Res 2006 34 Web Server issue 729 732 10.1093/nar/gkl320 PMC1538887 16845108

[B9] Goecks J Nekrutenko A Taylor J Team TG Galaxy: a comprehensive approach for supporting accessible, reproducible, and transparent computational research in the life sciences Genome Biol 2010 11 R86 10.1186/gb-2010-11-8-r86 20738864 PMC2945788

[B10] Deelman E Singh G Su M Blythe J Gil Y Kesselman C Mehta G Vahi K Berriman G Good J Laity A Jacob J Katz D Pegasus: a framework for mapping complex scientific workflows onto distributed systems Sci Programming 2005 13 219 237

[B11] Stevens R Tipney H Wroe C Oinn T Senger M Lord P Goble C Brass A Tassabehji M Exploring Williams-Beuren syndrome using myGrid Bioinformatics 2004 20 Suppl 1 i303 i310 10.1093/bioinformatics/bth944 15262813

[B12] Orvis J Crabtree J Galens K Gussman A Inman J Lee E Nampally S Riley D Sundaram J Felix V Whitty B Mahurkar A Wortman J White O Angiuoli S Ergatis: a web interface and scalable software system for bioinformatics workflows Bioinformatics 2010 26 12 1488 1492 10.1093/bioinformatics/btq167 20413634 PMC2881353

[B13] Goble CA Bhagat J Aleksejevs S Cruickshank D Michaelides D Newman D Borkum M Bechhofer S Roos M Li P Roure DD myExperiment: a repository and social network for the sharing of bioinformatics workflows Nucleic Acids Res 2010 38 2 W677 W682 20501605 10.1093/nar/gkq429 PMC2896080

[B14] Altintas I Berkley C Jaeger E Jones M Ludascher B Mock S Kepler: an extensible system for design and execution of scientific workflows Proceedings of the 16th International Conference on Scientific and Statistical Database Management 2004 Santorini Island Greece 423 424

[B15] Deelman E Gannon D Shields M Taylor I Workflows and e-Science: An overview of workflow system features and capabilities Future Gener Comput Syst 2009 25 5 528 540 10.1016/j.future.2008.06.012

[B16] Gentleman R Carey V Bates D Bolstad B Dettling M Dudoit S Ellis B Gautier L Ge Y Gentry J Hornik K Hothorn T Huber W Iacus S Irizarry R Leisch F Li C Maechler M Rossini A Sawitzki G Smith C Smyth G Tierney L Yang J Zhang J Bioconductor: open software development for computational biology and bioinformatics Genome Res 2004 5 R80 10.1186/gb-2004-5-10-r80 PMC545600 15461798

[B17] Excoffier L Heckel G Computer programs for population genetics data analysis: a survival guide Nat Rev Genet 2006 7 10 745 758 10.1038/nrg1904 16924258

[B18] Mueller L Brusniak M Mani D Aebersold R An assessment of software solutions for the analysis of mass spectrometry based quantitative proteomics data J Proteome Res 2008 7 51 61 10.1021/pr700758r 18173218

[B19] Machado M Magalhaes WCS Sene A Araujo B Faria-Campos A Chanock S Scott L Oliveira G Tarazona-Santos E Rodrigues MR Phred-Phrap package to analyses tools: a pipeline to facilitate population genetics re-sequencing studies Invest Genet 2011 2 3 10.1186/2041-2223-2-3 PMC3041995 21284835

[B20] Falush D Stephens M Pritchard J Inference of population structure using multilocus genotype data: linked loci and correlated allele frequencies Genetics 2003 164 1567 1587 12930761 10.1093/genetics/164.4.1567 PMC1462648

[B21] Li H Handsaker B Wysoker A Fennell T Ruan J Homer N Marth G Abecasis G Durbin R 1000 Genome Project Data Processing Subgroup The sequence alignment/map (SAM) format and SAMtools Bioinformatics 2009 25 2078 2079 10.1093/bioinformatics/btp352 19505943 PMC2723002

[B22] Rios J Karlsson J Trelles O Magallanes: a web services discovery and automatic workflow composition tool BMC Bioinformatics 2009 10 1 12 10.1186/1471-2105-10-1 19832968 PMC2771019

[B23] Lamprecht A Margaria T Steffen B Bio-jETI: a framework for semantic-based service composition BMC Bioinformatics 2009 10 1 19 10.1186/1471-2105-10-1 19796405 PMC2755829

[B24] Martin D Paolucci M McIlraith S Burstein M McDermott D McGuinness D Parsia B Payne T Sabou M Solanki M Bringing Semantics to Web Services: the OWL-S approach Lecture Notes Comput Sci 2005 3387 26 42 10.1007/978-3-540-30581-1_4

[B25] The World Wide Web Consortium Web Services Description Language (WSDL) 1.1. 2001 http://[http://www.w3.org/TR/wsdl]

