# Supplementary material for: A graph-based approach for designing extensible pipelines
Source: BMC Bioinformatics. 2012 Jul 12;13:163. doi: 10.1186/1471-2105-13-163 (PMC3496580; doi:10.1186/1471-2105-13-163)
Supplement: Additional file 1 — Supplementary Information. This document provides additional information on the performance measure, the pipeline system algorithm, the list of tools in the pipeline application, and the complete Tool Registry for the Format Conversion Pipeline. [file 1471-2105-13-163-S1.pdf]

A Graph-based Approach for Designing Extensible Pipelines (Supplementary Information)

Maíra R. Rodrigues [1,], Wagner C. S. Magalhães [1], Moara Machado [1] Eduardo Tarazona-Santos [1,*]
1 Departamento de Biologia Geral, Universidade Federal de Minas Gerais,
Av. Antonio Carlos 6627, Pampulha, Caixa Postal 486, 31270-910,
Belo Horizonte, Brazil.

February 2, 2012

1 The Performance Measure

The performance measure indicates the time that a tool takes to process one or more input files. It is used to identify the fastest pipeline, in case there are alternative pipelines for the same task. It is calculated as follows:

$performance\_measure = (time/filesizesum) * 10$

where $time$ is the time the tool took to execute (in seconds), and $filesizesum$ is the size of its input file (in KB) (or files, in case the tool requires extra input files). This evaluation must be performed by the pipeline system's administrator on the analysis server where the tools are executed to avoid a difference in performance due to distinct machine configurations. It is also recommended that the pipeline system's administrator keep sample input files for each supported data types and used this same set in all evaluations. For example, files $sdat1test$ and $sdat2test$ are used as input to evaluate all tools that accept the SDAT format as input format. This avoids a bias due to sample size. We provide a java program at the project's website (http://code.google.com/p/dynamic-pipeline/) to help calculating the performance measure.

2 Pipeline System Algorithm

The pipeline system algorithm is formalised in Algorithm 1. The function $createIODependencies()$ in line 16 is formalised in Algorithm 2.

3 List of Tools in the Dynamic Pipeline Application

Currently, our format conversion pipeline handles data formats compatible with the following software: PolyPhred (for polymorphism identification from aligned raw sequences reads) **?**, PHASE (to infer chromosome phase) **?**, DnaSP (for general population genetics analysis) **?**, Structure (for population structure inferences) **?**, Sweep (for natural selection inferences) **?**, Haploview (for linkage disequilibrium analysis) **?** and R-based tools for population genetics and genetic epidemiology such as HierFstat (for inferences about population structure) **?**. More information about these software programs can be found on their website listed in Table 1. The pipeline also handles general purpose file formats such as SDAT (Figure 1), NEXUS and PrettyBase.

Software	Website
FastPhase	http://stephenslab.uchicago.edu/software.html
Structure	http://pritch.bsd.uchicago.edu/software.html
Phase	http://stephenslab.uchicago.edu/software.html
PolyPhred	http://droog.gs.washington.edu/polyphred/
DnaSP	http://www.ub.edu/dnasp/
Sweep	http://www.broadinstitute.org/mpg/sweep/
Haploview	http://www.broadinstitute.org/scientific-community/science/programs/medical-and-population-genetics/haploview/haploview
R-HierFstat	http://www2.unil.ch/popgen/softwares/hierfstat.htm
R-Adegenet	http://adegenet.r-forge.r-project.org/

Table 1: List of websites for the software programs with data formats supported by our format conversion application.

Algorithm 1 Pipeline system algorithm.

input: $start, end, toolRegistry, wt$
while $Line_j$ in toolRegistry **do**
  $E_j(i, o, t, l, c, XI, XO, f, b, r, e)$ =parseEntry($Line_j$)
  if !containVertex($G, E_j[i]$) **then**
    addVertex($G, E_j[i]$)
  end if
  if !containVertex($G, E_j[o]$) **then**
    addVertex($G, E_j[o]$)
  end if
  if wt =“performance” **then**
    $w_j = E_j[f]$
  else if $wt =$ “dependencies” **then**
    w_j =length($E_j[XI]$)
  end if
  addEdge($G, E_j[i], E_j[o], E_j[t], w_j$)
  createIODependencies($G, E_j[i], E_j[o], E_j[t], E_j[XI], E_j[XO]$)
end while
$P =$ findToolPath($start, end, G$)
$previousTarget_n = null$
for all $t_n \in P$ **do**
  $index = 0$
  $tgt_n =$ edgeTarget(t_n)
  $IE_{tgt_n} =$ incomingEdges(tgt_n)
  $SIE_{tgt_n} = \{(t_1, ..., t_k) : IE_{tgt_n} | t_n = t_k\}$
  for all $t_m \in SIE_{tgt_n}$ **do**
    $t_m =$ edgeSource(tgt_m)
    if $src_m \neq previousTarget_n$ **then**
      $LI[index] = src_m$
      $index = index + +$
    end if
  end for
  $previousTarget_n = tgt_n$
end for
$LO =$ findOutputs(P)
(.inputs,.exec,.outputs) = writeExecutables(LI, P, E, LO)

4 Complete Tool Registry for the Format Conversion Pipeline

The complete Tool Registry for the Format Conversion pipeline application is presented in Table 1. Each line contains information about one conversion tool that composes the pipeline system. The tool registry was instantiated by both the tools' providers and the system administrator on request from the providers. A different application will have its own tool registry, instantiated with the appropriate tools for that specific application.

Algorithm 2 Algorithm for the function $createIODependencies()$. It adds the scripts' input and output dependencies to the Graph G.

input: G, i, o, t, XI, XO
$ew = MINWEIGHT$
if $|XI| \geq 1$ **then**
    for all $d_k \in XI$ **do**
        if containVertex(G, d_k) **then**
            $d_k^n = d_k$
            while containVertex(G, d_k^n) **do**
                $d_k^{n+1} = d_k$
            end while
            addVertex(G, d_k^n)
            addEdge(G, d_k^n, o, t, w)
        else
            addVertex(G, d_k)
            addEdge(G, d_k, o, t, w)
        end if
    end for
end if
if $|XO| \geq 1$ **then**
    for all $p_k \in XO$ **do**
        if containVertex(G, p_k) **then**
            $p_k^n = p_k$
            while containVertex(G, p_k^n) **do**
                $p_k^{n+1} = p_k$
            end while
            addVertex(G, p_k^n)
            addEdge(G, i, p_k^n, t, w)
        else
            addVertex(G, p_k)
            addEdge(G, i, p_k, t, w)
        end if
    end for
end if

SDAT Format

	1369	1572	1709	1715	1756	1778	1867	1908	1961	1992
NCP001	TC	GG	GG	GG	GG	AG	GG	TT	GG	AG
NCP002	TT	GG	GG	GG	GG	AA	GG	TT	GG	AA
NCP003	TT	GG	GG	GG	GG	AA	GG	TT	GG	AA
NCP004	TT	GG	GG	GG	GG	AG	GG	TT	GG	AG
NCP005	TT	GG	GG	GG	GG	AG	GG	TT	GG	AG
NCP006	TT	GG	GG	GG	GG	GG	GG	TT	GG	GG
NCP007	TT	GG	GG	GG	GG	GG	GG	TT	GG	GG
NCP008	TT	GG	GG	GG	GG	AA	GG	TT	GG	AA
NCP009	TT	GG	GG	GG	GG	AG	GG	TT	GG	AG
NCP010	CC	GG	GG	GG	GG	GG	GG	??	??	??
NCP011	TT	GG	GG	GG	GG	AG	GG	TT	GG	AG
NCP012	TC	GG	GG	GG	GG	GG	GG	TT	GG	GG
NCP013	TC	GG	GG	GG	GG	AG	GG	TT	GG	AG
NCP014	TT	??	??	??	??	??	GG	TT	GG	AG
NCP015	TT	GG	GG	GG	GG	AG	GG	TT	GG	AG
NCP016	TT	GG	GG	AG	GG	AG	GG	TT	AG	AG

The first line must have the allele positions separated by blank spaces.

Other lines:

1 - Individuals name
2 - Alleles for each position declared on first line

Figure 1: Sample SDAT-formatted data file.

INPUT	OUTPUT	SCRIPT	LANGUAGE	CODE	DEPENDENCIES	SUBPRODUCTS	PERFORMANCE	OBSERVATIONS	PROVIDER	CONTACT
POLY_OUT	PrettyBase	PolyPhred2PrettyBase.pl	perl	1	-	-	0.0048	-	Wagner Magalhaes	@
PrettyBase	SDAT	PrettyBase2SDAT.pl	perl	2	-	-	0.0117	-	Wagner Magalhaes	@
SDAT	PrettyBase	SDAT2PrettyBase.pl	perl	3	-	-	0.0200	-	Wagner Magalhaes	@
SDAT	PHASE_Format	SDAT2Phase.pl	perl	4	-	-	0.0267	-	Wagner Magalhaes	@
SDAT	Structure_Format	SDAT2Structure.pl	perl	5	-	mainparam,extraparam	0.1471	-	Wagner Magalhaes	@
SDAT	NEXUS	SDAT2NEXUS.pl	perl	6	-	-	0.1586	-	Giordano Bruno	@
SDAT	R_Hierfstat	SDAT2Rhierfstat.pl	perl	7	SDAT	-	0.0250	Each fastPHASE file corresponds to a population and only one population file is mandatory. When using two or more files, the number of loci must be the same in all files.	Wagner Magalhaes and Giordano Bruno	@
SDAT	R_Genetics	SDAT2Rgenetics.pl	perl	8	SDAT	-	2.1050	Each SDAT file corresponds to a population and only one population file is mandatory. When using two or more files, the number of loci must be the same in all files.	Wagner Magalhaes and Giordano Bruno	@
PHASE_OUT	Fasta	Phase2Fasta.pl	perl	9	Fragments,RefSeq	-	0.0163	-	Wagner Magalhaes	@
PHASE_OUT	Haploview_Format	Phase2Haploview.pl	perl	10	-	-	0.0048	-	Wagner Magalhaes	@
PHASE_OUT	Sweep_Format	Phase2Sweep.pl	perl	11	-	-	0.0048	-	Wagner Magalhaes	@
FPHASE_OUT	R_Hierfstat	FastPhase2Rhierfstat.pl	perl	12	FPHASE_OUT	-	0.0269	Each fastPHASE file corresponds to a population and only one population file is mandatory. When using two or more files, the number of loci must be the same in all files.	Wagner Magalhaes	@
FPHASE_OUT	R_Genetics	FastPhase2Rgenetics.pl	perl	13	FPHASE_OUT	-	0.0192	Each fastPHASE file corresponds to a population and only one population file is mandatory. When using two or more files, the number of loci must be the same in all files.	Wagner Magalhaes	@
POLY_OUT	Fasta	PolyPhred2Fasta.pl	perl	14	RefSeq	-	6.2951	-	Wagner Magalhaes	@
NEXUS	R_Hierfstat	NEXUS2Rhierfstat.pl	perl	15	NEXUS	-	0.0259	Each NEXUS file corresponds to a population and only one population file is mandatory. When using two or more files, the number of loci must be the same in all files.	Wagner Magalhaes	@

Table 2: Complete tool registry for the format conversion pipeline.
